# An atypical case report of an arrhythmogenic cardiomyopathy in a 70-years-old patient with suggestive left ventricular signs

**DOI:** 10.1016/j.radcr.2024.03.082

**Published:** 2024-05-10

**Authors:** ikram Tahani, oumayma Hattab, Nabila ismaili, Noha el ouafi

**Affiliations:** Department of Cardiology, Mohammed VI University Hospital of Oujda, Faculty of Medicine and Pharmacy, Mohammed the First University of Oujda, Morocco

**Keywords:** Arythmogenic ventricular cardiomyopathy, Arythmias, Arythmogenic right ventricule cardiomyopathy, Ventricular tachycardia, MRI, Epsilon waves, Cardiac defibrilator

## Abstract

Arrhythmogenic dysplasia of the right ventricule (ARVC), actually known as arrhythmogenic cardiomyopathy (ACM) is a rare genetic condition caused by the replacement of the normal right ventricular myocardium with fibrofatty tissue. However, 2 other phenotypes affecting the left ventricle were recently discovered. The disease usually appears in patients ranging from 30 to 50 years old; in fact, about 80% of cases occur in young patients <40 years of age. Therefore, it is usually considered in young adults or athletes presenting with a history of syncope, ventricular arrhythmias (VA), and/or sudden cardiac death (SCD). We report an atypical case of a 70-year-old male who was admitted to the hospital for spontaneous ventricular tachycardia (VT) that was reduced by an immediate electric shock, and the paraclinical investigations strongly supported the presence of an almost complete form of the disease with electric signs in favor of possible left ventricular (LV) damage, which makes the case even more interesting.

## Introduction

Considered a serious disease due to the life-threatening circumstances in which it can occur, ACM is the most frequent cause of sudden death in young adults, followed by hypertrophic cardiomyopathy, and is responsible for 30% of deaths in young adults and 5% in subjects over 65 years [1]. It causes a real diagnosis problem because of its varied and non-specific presentations. Classically, it is a pathology that mainly affects the RV (right ventricule) except for several cases of biventricular and left-dominant involvement that have recently led to the proposed term 'arrhythmogenic ventricular cardiomyopathy' [2]. We present the case of a 70-year-old patient who has presented with VT on 3 occasions with the stigmata of biventricular involvement.

## Case description

Our patient presented to the emergency department with sudden onset palpitations associated with profuse sweating; symptomatology similar to previous episodes with a background of chronic effort dyspnea. Initial clinical findings revealed an unstable conscious patient with blood pressure at 70/40 mmHg, a heart rate (HR) of 120 beats per minute, and cold and mottled extremities.

He already suffers from a metabolic syndrome consisting of hypertension on well-balanced triple therapy, diabetes, and abdominal obesity, and was a known smoker with no family history of sudden death or extreme sport but a history of 2 episodes of undocumented VT; the first dating back 3 years ago and the second 2 years later reduced by external electric shock in another care facility, according to the patient. Cardiovascular and pulmonary examinations were not much helpful, apart from the presence of oedema of the lower limbs reaching mid-leg.

**Fig. 1:**
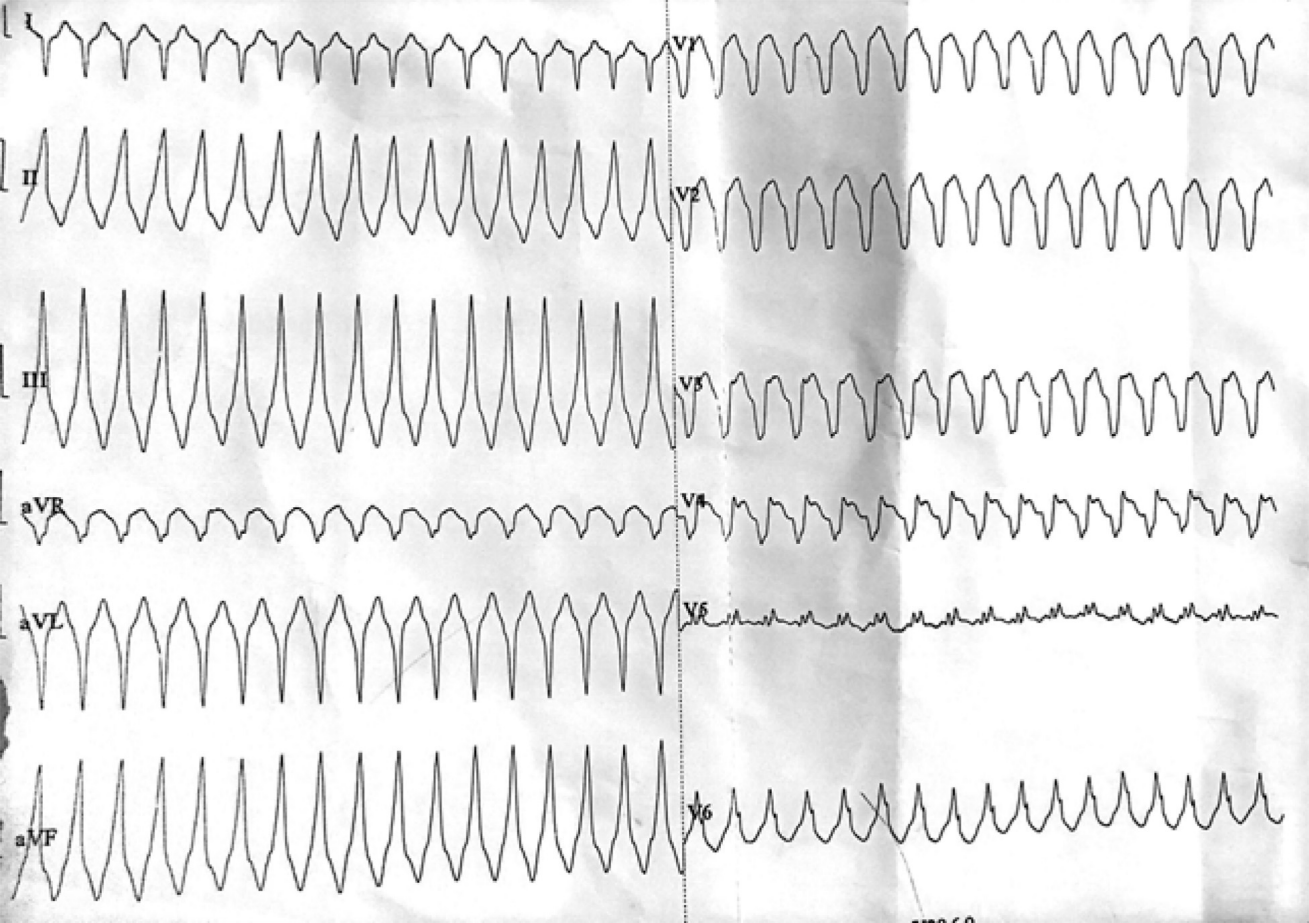
Emergency EKG showing ventricular tachycardia.

**Fig. 2:**
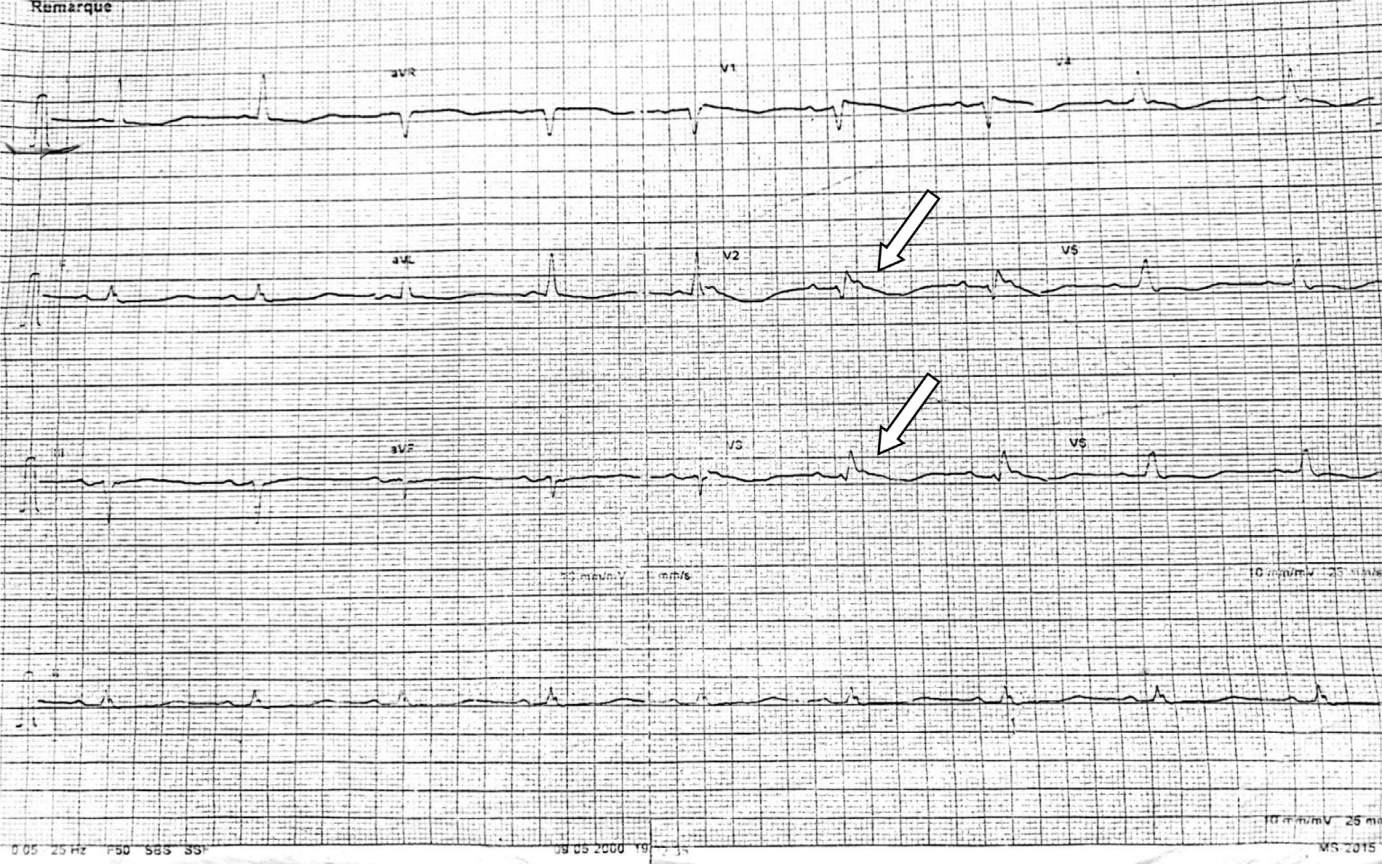
Post electric shock EKG in sinusal rythm showing incomplete RBBB, epsilon waves (white arrows) in V2, V3 and negative T waves in anteroseptoapical leads.

**Fig. 3:**
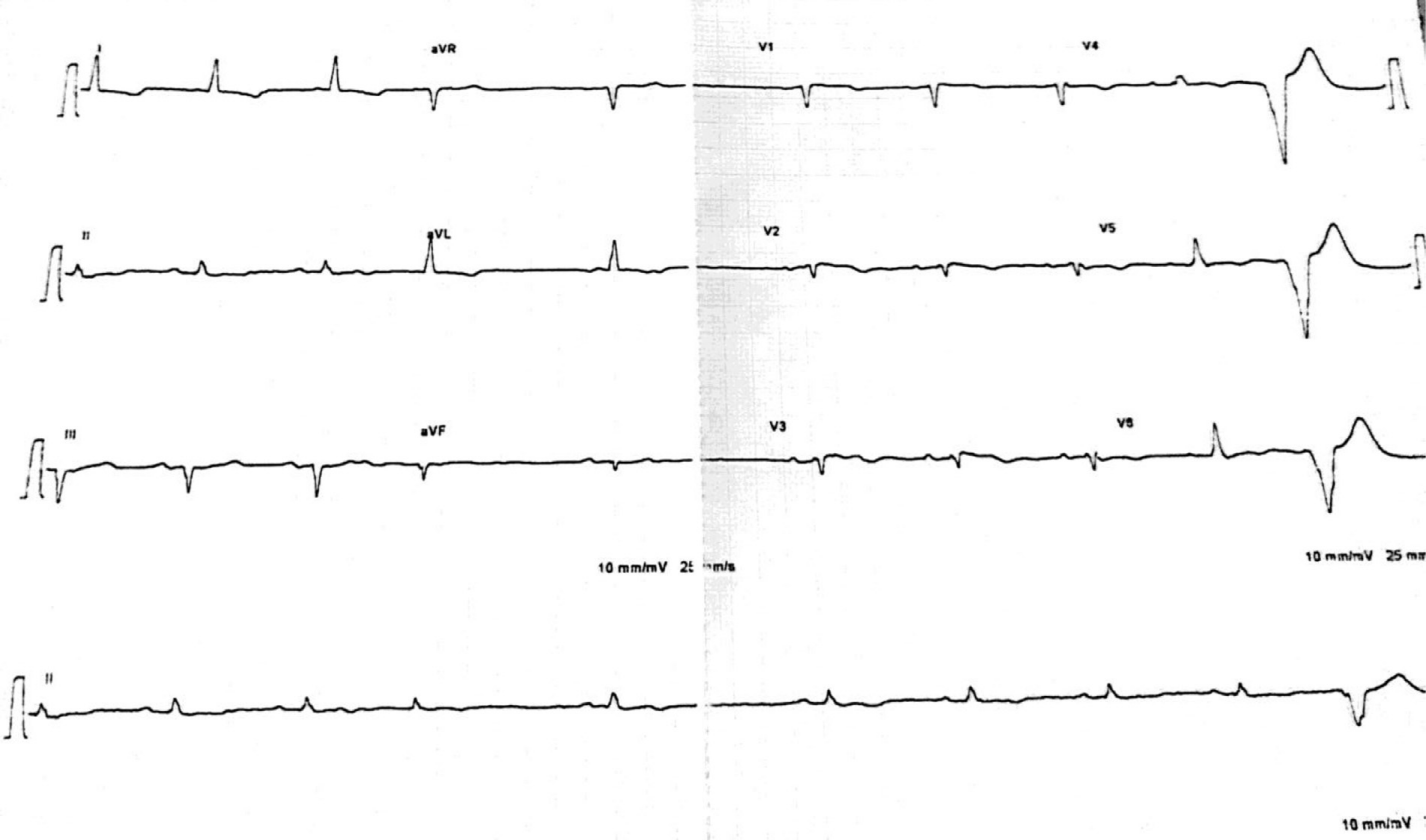
An old EKG of the patient in sinusal rythm showing no epsilon waves but ventricular extrasystoles.

An immediate electrocardiogram (ECG) revealed tachycardia with wide QRS complexes beating at a rate of 204 per minute with atrioventricular dissocation (Fig. 1). The VT score was calculated at 4, in favor of a lower-axis VT (positive QRS in DII and DIII) originating from the left upper quadrant (negative QRS in V1), more precisely from the RV outflow tract and right coronary cusp (QS appearance in V1 and transition in V5-V6). A first electric shock was immediately performed in vain, and it was the second one that reduced the rhythm disturbance, after which the patient was put on maintenance cordarone. The ECG in sinus rhythm revealed a left heart axis, an incomplete right bundle block (RBB), and epsilon waves following the QRS in V2, V3, as well as negative T waves in the anteroseptoapical leads (Fig. 2). Interestingly, there were no epsilon waves on an old ECG of the same patient dating back 1 year ago (Fig. 3), supporting the concept that ACM is generally progressive in nature and that a surface 12-lead ECG is able to assess disease progression.

**Fig. 4:**
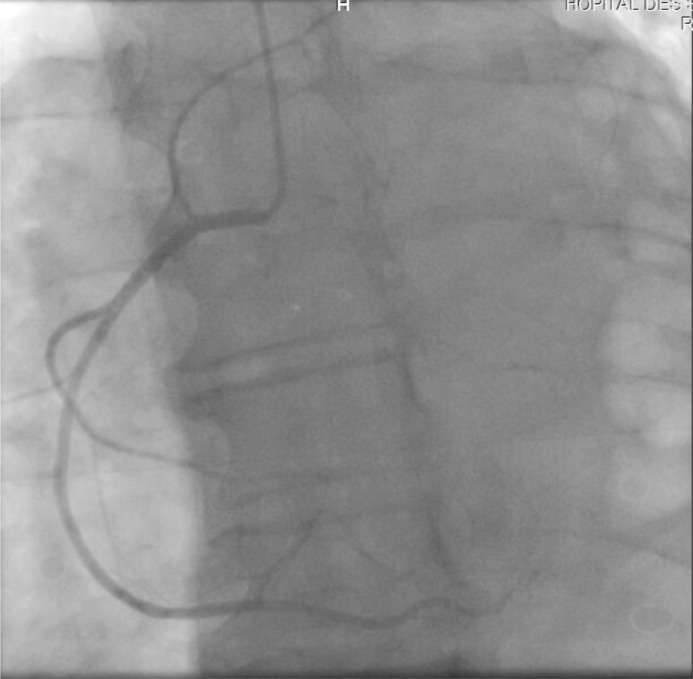
coronary angiography image showing a right coronary artery angiographically normal.

**Fig. 5:**
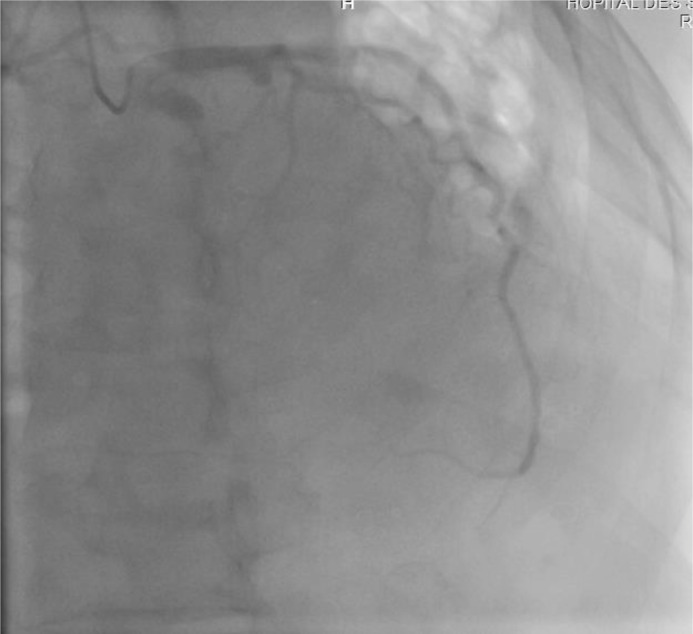
coronary angiography image showing a left coronary network angiographically normal.

Echocardiography showed an enlarged RV in severe systolic dysfunction with a trabuculated apex and a tricuspid annulus dilated to 43 mm. The left ventricle was hypertrophied and non dilated, with good systolic function : LVEF (left ventricular ejection fraction) at 65%. Ventricular hyperexcitability was recorded, and late ventricular potentials (LVP) were not performed as they were not available in our training center. The coronary network was angiographically normal (Figs. 4 and 5), pleading in favor of the non-ischemic origin of the rhythm disorder. Biologically, the thyroid work-up and blood ionnogram were correct, and troponins were slightly elevated, probably linked to myocardial sideration, given that they were performed after electric shock. The BNP assay came back elevated at 909 pg/mL.

**Fig. 6:**
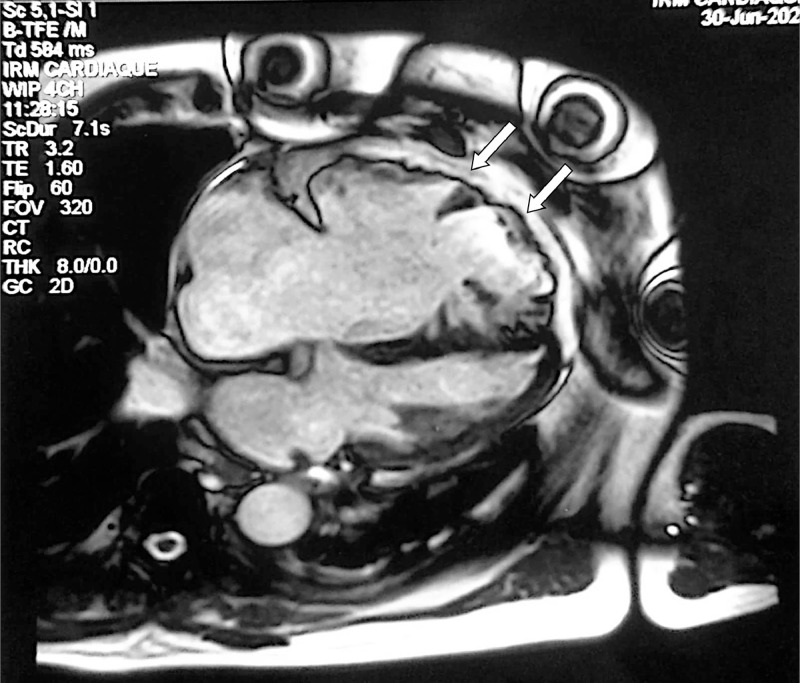
Cardiac MRI cine-MRI sequence incidence 4 cavities showing trabeculations of the apex of the RV (white arrows).

**Fig. 7:**
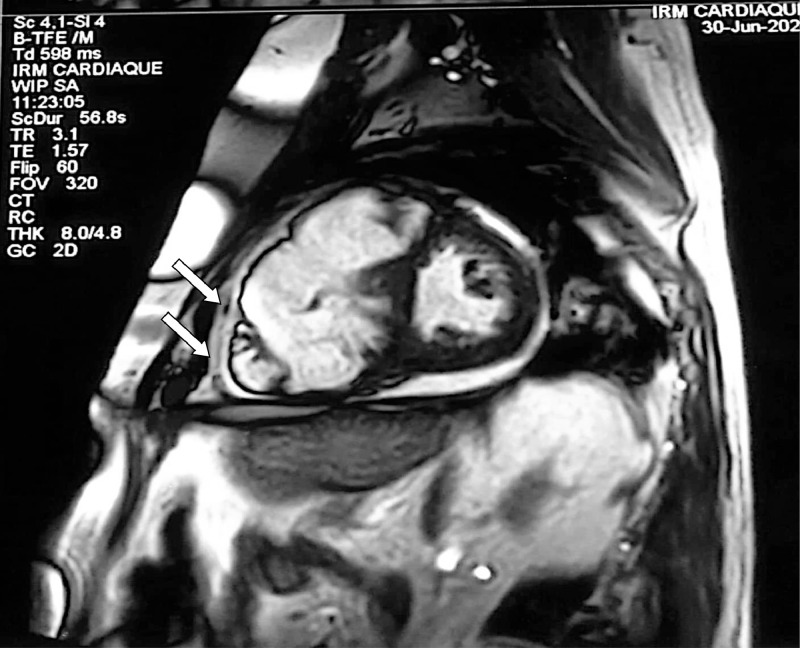
Cardiac MRI sequence Cine-MRI short-axis sequence showing RV dilatation with trabeculations of the apex of the RV (white arrow).

Cardiac magnetic resonance (CMR) confirmed the diagnosis, showing a highly dilated RV with an end-diastolic volume of 402 mL, equal to 191 mL indexed (Fig. 7), with segmental kinetic disorders of the RV, notably areas of hypokinesis of the free wall, several areas of dyskinesia, and bulging of the inferior and anterior wall of the RV, which is dysfunctional, with an RVEF (right ventricular ejection fraction) of 55% (overestimated due to the massive tricuspid regurgitant insufficiency). MRI also revealed hypertrabeculation of the apex (Fig. 6) and hypertrophy of the moderator band. The tricuspid valves presented a coaptation defect, with dilatation of the annulus and tricuspid leakage, most probably massive and laminar. There were no signs of myocardial necrosis.

The 2010 International Task Force Criteria (Table 1) are divided into major and minor categories. The “definitive” diagnosis of ARVC is made by the inclusion of 2 major criteria, 1 major and 2 minor criteria, or 4 minor criteria. The “borderline” diagnosis includes 1 major and 1 minor criterion, or 3 minor criteria from different categories. “Possible” ARVC includes 1 major criterion or 2 minor criteria. For our patient, the diagnosis was confirmed on a score of 7 according to this last criterion: (3 major criterions rated at 2 each: segmental dysfunction with structural abnormalities of the RV on MRI, depolarization abnormalities including the epsilon waves, and repolarization abnormalities. 1 minor criterion which is arrhythmias). The sum is greater than 4, making arrhythmogenic cardiomyopathy a certain diagnosis for this patient.

**Table 1:** Updated 2010 diagnostic criteria : Adapted from Augusto et al [18].

1. Global or regional dysfunction and structural alterations :
Major
*At the two-dimensional echocardiogram:* - Regional right ventricular akinesis, dyskinesis or aneurysm And one of the following (end-diastolic): - RVOT PLAX ≥ 32 mm (corrected for the body surface area − PLAX/BSA ≥ 19 mm/m2) - RVOT PSAX ≥ 36 mm (corrected for the body surface area − PLAX/BSA ≥ 21 mm/m2)
- Or fractional alteration of the area ≤ 33%
*At the MRI:* - Regional right ventricular akinesis, dyskinesis or dys-synchrony in right ventricular contractions And one of the following :
Ratio of right ventricular end-diastolic volume and BSA ≥110 mL/m^2^ (male) or BSA ≥100 mL/m^2^ (female) – Or right ventricular ejection fraction ≤ 40%
*At the right ventricular angiography*: - Right ventricular regional akinesis, dyskinesis or aneurysm
Minor
*At the two-dimensional echocardiogram:* - Right ventricular regional akinesis or dyskinesis And one of the following (end diastolic): - RVOT PLAX ≥29 mm and <32 mm (corrected for the body surface area − PLAX/BSA ≥16 mm/m^2^ and <19 mm/m^2^) - RVOT PSAX ≥32 mm and <36 mm (corrected for the body surface area − PLAX/BSA ≥ 18 mm/m2 and < 21 mm/m2) - Or fractional alteration of the area > 33% and ≤ 40%
*At the MRI*: - Regional right ventricular akinesis or dyskinesis or dys-synchrony in right ventricular contraction And one of the following:
- Ratio of right ventricular end-diastolic volume and BSA ≥100 and <110 mL/m^2^ (man) or ≥90 and <100 mL/m^2^ (woman) - Or Right ventricular ejection fraction >40% and ≤45%
2. Histological characterization of the ventricular wall :
Major
- Residual myocytes <60% in the morphometric analysis (or <50% by estimation) with fibrous replacement of the right ventricular free wall in more than one sample, with or without adipose replacement in myocardial biopsy
Minor
- 60-75% of residual myocytes in the morphometric analysis (or 50%-65% by estimation) with fibrous replacement of right ventricular free wall in more than one sample, with or without adipose replacement in myocardial biopsy
3. Ventricular repolarization alterations :
Major - Inverted T waves in V1, V2, or V3 in individuals aged >14 years in the absence of complete RBBB with QRS ≥ 120 ms
Minor
- Inverted T waves in V1 and V2 in individuals aged > 14 years in the absence of complete RBBB with QRS ≥ 120 ms or in V4, V5 or V6
Inverted T waves in V1, V2, V3 and V4 in subjects aged > 14 years in the presence of complete RBBB with QRS ≥ 120 ms
4. Conduction/depolarization alterations :
Major - Epsilon wave in leads V1 to V3
Minor
- Duration of QRS terminal activation ≥55 ms measured from the S wave nadir to the end of QRS, including R’ at V1, V2, V3 in the absence of complete RBBB of the His bundle
- High-resolution ECG late potentials in more than one of the following three parameters in the absence of QRS ≥ 110 ms on the standard 12-lead ECG:
- Duration of filtered QRS (fQRS) ≥114 ms
- QRS terminal duration <40 μV (low amplitude signal duration) ≥38 ms
- Root mean square of the potential in the 40 ms terminals of ventricular activation (MRIs40 - mV) ≤20 μV
5. Arrhythmias :
Major
- Sustained or n-sustained ventricular tachycardia with complete LBBB morphology with superior axis (QRS negative or undetermined in II, III, aVF, and positive in aVL)
Minor
- Sustained or n-sustained ventricular tachycardia with right ventricular outflow tract configuration, complete LBBB morphology with inferior axis (QRS positive in II, III and aVF and negative in aVL) or of indeterminate axis
- >500 ventricular extrasystoles in the 24-hr Holter monitoring
6. Family history :
Major
- Confirmed ARVD in a first-degree relative meeting the Task Force criteria
- ARVD confirmed by histopathology at the autopsy or surgery in first-degree relative
- Identification of pathogenic mutation categorized as associated or likely to be associated with ARVD* in a patient undergoing Evaluation
Minor
- History of ARVD in a first-degree relative in whom it is not possible or the feasibility of confirming the presence of Task Force criteria is difficult
- Sudden cardiac death (age <35 years) due to suspected ARVD in first-degree relative
- ARVD confirmed by histopathology or according to the current Task Force criteria in second-degree relative
Two major, or 1 major and 2 minor, or 4 minor criteria: definite diagnosis of AC. One major and 1 minor, or 3 minor criteria: borderline diagnosis; 1 major, or 2 minor criteria from different categories: possible diagnosis

Our patient was managed with rate-control medications (betablockers) and an antiarrhythmic agent (amiodarone), as well as the combination of angiotensin-converting enzyme (ACE) inhibitors, calcium channel blockers (CCB) and spirolactone for his heart failure and hypertension. While insisting on a low-salt diet of maximum 6 g per day, water restriction of 1500 mL per day, and avoidance of high-intensity exercise as indicated in the 2022 recommendations for ventricular arrhythmias [2]. Implantation of an automatic defibrillator could not be carried out, however, as it was not available in our training unit. For genetic counseling, the patient had no descendants, and no rythmic events were recorded after 1 year, nor were any palpitations reported.

## Discussion

**Fig. 8:**
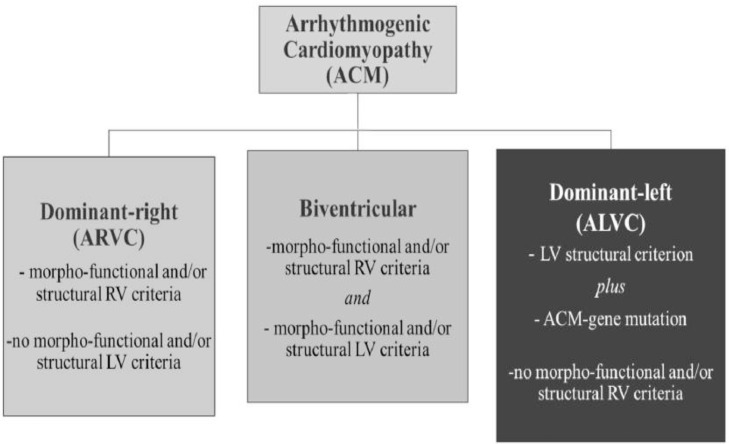
Diagnostic criteria for the various phenotypes of arrhythmogenic cardiomyopathy : Adapted from corrado et al [4].

It was in 1977 that Guy Fontaine, a French cardiologist, and electrophysiologist, described arrhythmogenic cardiomyopathy, which is characterized by a fibrofatty replacement of the heart muscles [3], predominantly affecting the RV. However, there are several phenotypes that classify the disease into the RV phenotype, the LV phenotype and the biventricular one (Fig. 8) [4]. The severity of the disease is his tendency to generate spontaneous malignant cardiac arrhythmias that could lead to sudden death. The prevalence ranges from 1:1000 to 1:5000 individuals, with male predominance among probands [5]. Furthermore, the true prevalence of the disease may be higher since clinical and postmortem examinations may miss the diagnosis. Unlike in our case, most cases manifest during the fourth decade of life. No occupation has been documented as its predisposition; rather, it is ted that intense exercise is probably a trigger of its “early” manifestation, as sudden cardiac death and eventual post-mortem diagnoses are more often seen among young athletes [6]. As for smoking, several studies have shown that it increases the severity of all types of arrhythmias [7,8]. Our patient was a smoker shoemaker and was not an athlete.

The disease has been described as autosomal dominant with variable penetrance [3]. A rare autosomal recessive variant has been described, which is associated with woolly hair and palmoplantar keratoderma (Naxos disease). As a matter of fact, ACM is caused by pathogenic mutations in desmosomal genes and less commonly in non-desmosomal genes, both responsible for abnormal proteins leading to myocyte detachment, increased activity of enhanced adipogenesis, fibrogenesis causing thining of trabeculae, aneuvrysmal dilatation, and myocyte apoptosis [9]. These transformations are further enhanced by factors increasing wall stress, such as exercise, as a significant risk factor [9]. Genetic testing is indicated, and the identification of a mutation (in up to 73% of probands) is a major criterion for the diagnosis. About 116,671 compound/ digenic mutations found in 4%-16% of cases have been associated with an increased risk of VA at a younger age [10]. In desmosome genetic analysis for the Han Chinese group study, 21 mutations were identified from 48 cases, and PKP2 was the most common mutated gene [11]. The disease penetrance in first-degree relatives is 28%-58%, supporting regular clinical evaluation of relatives [2].

ACM can present with 2 types of clinical aspects, both seen in our clinical case : ventricular arrhythmias such as ventricular fibrillation (VF), VT, sustained monomorphic or polymorphic VT, and cardiac failure signs such as dyspnea, asthenia, and oedema of the lower limbs. Studies have shown that patients with SCD at first presentation are typically younger (median age of 23 years), probably due to the compound / digenic mutations described above compared to those who present VTs (median age of 36 years) [10].

Palpitation is the most common symptom, manifesting in 27% to 67% of the cases [3], and occurs due to ventricular arrhythmias. It was the main symptom for our patient. Several functional signs are associated with the pathology, notably syncope (an alarming but inconsistent sign), dyspnea, and atypical chest pain [3]. The latter was ted in all 23 cases studied by Bariani et al [12], with at least one episode described in 90% of them, as well as in a 26-year-old Afro-American patient who described chest pain lasting up to 6 weeks [3]. It is very important to note that many patients with ACM, particularly those with sporadic cases, remain clinically asymptomatic for decades, making the condition difficult to diagnose [9]. Our patient presented with exertional effort dyspnea, but never complained of chest pain.

For paraclinical findings, more than 90% of ACM patients present EKG abnormalities [13]. A study by Jain et al. revealed that T waves inversions through V3 with the absence of a complete right bundle block (RBB) or the presence of an incomplete RBB pattern is the single best optimally sensitive and specific finding on EKG [9].

Interestingly, our patient showed electrical signs in favor of possible LV damage, notably the inversion of T waves in the low lateral position (V4 - V6), considered a minor Padua criterion; a more recent diagnostic criteria (Table 2) that takes into cognizance the involvement of the two ventricular compartments with 6 main categories : morpho-functional ventricular abnormalities, structural myocardial abnormalities, repolarization abnormalities, depolarization abnormalities, ventricular arrhythmias, and genetics. The right delay aspect of certain ventricular extrasystoles (absence of RS aspect in V4, V5, V6 and QR aspect in V6) is also in favor of LV damage that may be unseen yet at imaging (Fig. 3). Indeed, macroscopic or histologic involvement of the LV was found in 76% of hearts with ACM, and the latter was associated with clinical arrhythmic events, more severe cardiomegaly, inflammatory infiltrates, and heart failure [14].

**Table 2:** Padua criteria : Adapted from corrado et al [4].

For a long time considered a major MRI diagnostic sign, the detection of intramyocardial fat is now considered less reliable, given that its deposits are found in 15% of cases in normal subjects and up to 50% in overweight subjects [15]. Hypertrabeculations, found in 40% of cases, are not specific to ACM and may be seen in any pathology where right ventricular hypertrophy or dilatation exists. Only 3 types of MRI abnormalities, all of which were found in our patient, were selected as criteria for a positive diagnosis of ACM: segmental disorders of RV kinetics, reduced ejection fraction, and ventricular dilatation. The latter has a sensitivity of 77% and a specificity of 95%-100% for the diagnosis of ACM [15].

Kinetic disorders to look for include [7]: hypokinesia, akinesia, dyskinesia and telediastolic bulging; corresponding to a dyskinetic zone in systole and aneurysmal in diastole. The combination of the 2 is more specific for ACM than either alone [7]. Specificity is 100% when there is segmental dilatation of the RV opposite these dyskinetic zones. Late myocardial enhancement, missing in our patient, corresponds to areas of fibrosis, inflammation or oedema, within which the extracellular volume is increased.

Restriction from high-intensity exercise is regarded as a preventive tool in clinically affected ACM patients to reduce the risk of VAs and disease progression [2]. Management strategy also involves pharmacologic therapy, especially amiodarone with or without beta-blockers, which is considered the most effective drug combining the synergistic effect of class 3 antiarrhythmics with beta-blockers [16], to note that sotalol has the same success rate [9]. Radiofrequency ablation of an arrhythmogenic focus for patients who are unresponsive or intolerant to antiarrhythmic drugs is frequently unsuccessful and may require multiple attempts [17].

Once the diagnosis is made, an ICD (implantable cardiac defibrillator) is indicated for patients with ventricular fibrillation or non tolerated VT (class I) as well as for patients with well-tolerated sustained monomorphic VT (class IIa), according to 2022 ESC recommendations.

For primary prevention, ICDs should be considered in severe LV or RV dysfunction (IIa), a priori rythmic syncope (IIa), or moderate ventricular dysfunction associated with non-sustained VT or positive programmed ventricular stimulation (SVP)(IIa) [2]. Finally, heart transplantation is the last therapeutic option for ACM patients who have refractory arrhythmias or heart failure. Recommendations also insist on the realization of genetic studies in patients with a diagnosed or even suspected ACM [2]. This could not be realized in this patient since he has no decendence.

## Conclusion

Arrhythmogenic ventricular cardiomyopathy is a rare inherited cardiac muscle disease and should be considered as a possible cause in young patients presenting with ventricular tachyarrhythmias, as they are at risk of sudden death if not appropriately managed. It is important for physicians to be aware of the possibility of the disease in old adults who may present with palpitations, fatigue, and syncope where trigger disease (effort, sports) may be absent, making the diagnosis a real challenge.

## Patient consent

I assure that the patient has given his written informed consent for the relating to the subject matter above (“An atypical case report of an arrhythmogenic cardiomyopathy in a 70-years-old patient with suggestive left ventricular signs:”) to appear in a journal article, or to be used for the purpose of a thesis or presentation.
